# Recombinant HA-based vaccine outperforms split and subunit vaccines in elicitation of influenza-specific CD4 T cells and CD4 T cell-dependent antibody responses in humans

**DOI:** 10.1038/s41541-020-00227-x

**Published:** 2020-08-26

**Authors:** K. A. Richards, S. Moritzky, I. Shannon, T. Fitzgerald, H. Yang, A. Branche, D. J. Topham, J. J. Treanor, J. Nayak, Andrea J. Sant

**Affiliations:** 1 David H. Smith Center for Vaccine Biology and Immunology, Department of Microbiology and Immunology, University of Rochester Medical Center, 601 Elmwood Ave, Box 609, Rochester, NY 14642 USA; 2 Department of Pediatrics, Division of Infectious Diseases, University of Rochester Medical Center, 601 Elmwood Ave, Box 690, Rochester, NY 14642 USA; 3 Department of Biostatistics and Computational Biology, University of Rochester Medical Center, 265 Crittenden Blvd., Box 630, Rochester, NY 14642 USA; 4 Department of Medicine, Division of Infectious Diseases, University of Rochester Medical Center, 601 Elmwood Ave., Box 689, Rochester, NY 14642 USA

**Keywords:** Immunology, Vaccines

## Abstract

Although traditional egg-based inactivated influenza vaccines can protect against infection, there have been significant efforts to develop improved formats to overcome disadvantages of this platform. Here, we have assessed human CD4 T cell responses to a traditional egg-based influenza vaccine with recently available cell-derived vaccines and recombinant baculovirus-derived vaccines. Adults were administered either egg-derived Fluzone^®^, mammalian cell-derived Flucelvax^®^ or recombinant HA (Flublok^®^). CD4 T cell responses to each HA protein were assessed by cytokine EliSpot and intracellular staining assays. The specificity and magnitude of antibody responses were quantified by ELISA and HAI assays. By all criteria, Flublok vaccine exhibited superior performance in eliciting both CD4 T cell responses and HA-specific antibody responses, whether measured by mean response magnitude or percent of responders. Although the mechanism(s) underlying this advantage is not yet clear, it is likely that both qualitative and quantitative features of the vaccines impact the response.

## Introduction

Influenza vaccine practices and production methods have evolved greatly in past decades (reviewed in refs ^1,2^). Although early strategies involved inactivated whole virus vaccines, issues of reactogenicity prompted development of vaccines made from extracts from virions grown in embryonated chicken eggs. The production methods for egg-based vaccines have been modified through the years, and now typically involve virion inactivation, detergent or ether extraction (or “splitting”) and subsequent steps to enrich for hemagglutinin (HA). These procedures are typically proprietary with each vaccine manufacturer. Generally, inactivated influenza vaccines can be characterized as “split” or “subunit” based on the extent of removal of the internal virion proteins, with the primary goal being enrichment for the HA proteins, the target of vaccination aimed at elicitation of neutralizing antibodies.

Although many studies have shown the effectiveness of inactivated influenza vaccines (“IIV”), both in terms of protection from infection and in eliciting protective antibody responses (reviewed in refs ^3,4^), there are several problematic features of egg-based vaccines that have prompted interest in development of new production strategies (reviewed in refs ^5,6^). The first disadvantage is that they rely on an adequate supply of chicken eggs, which may be limiting in the times of a pandemic threat. Also, production schedules are long and depend on adaptation of human isolates of influenza virus for growth in eggs. A more serious issue, noted many years ago, is that the adaptation of the influenza virus to growth in eggs can cause amino acid substitutions in the HA that can alter or eliminate dominant antigenic sites on HA^7–9^. Finally, production of virions in eggs leads to HA glycosylation patterns that are distinct quantitatively and qualitatively from those expressed in mammalian cells, potentially altering HA immunogenicity or masking particular protection-associated antigenic sites on the HA protein.

Because of these difficulties in production and immunogenicity of egg-based vaccines, recent efforts have sought to develop alternative platforms (reviewed in ref. ^10^). Two licensed vaccines became available recently that have potential advantages in vaccine production and efficacy. One of these involves a mammalian cell-based vaccine production system^11^, where influenza viruses are expanded in a cultured mammalian cell line. This platform offers at least two advantages. First, the cultured cells used for production can be expanded and available for use within short and predictable timelines. Also, because virions are produced in a mammalian system, large-scale production is less likely to lead to mutations of influenza HA and NA genes. A second recently developed influenza vaccine is based on a baculovirus HA expression system^12,13^. This system allows exclusive production of recombinant HA proteins, as soon as the strains have been identified and sequenced. The individual HA proteins are extracted from the insect cell line, purified and then combined into multivalent vaccines with no other source of non-HA influenza virus-derived proteins.

In this study, we performed a comprehensive study of the human CD4 T-cell response to vaccination with these three distinct types of licensed influenza vaccines. We have compared immunity induced by the egg-based split vaccine Fluzone^14^, the mammalian cell-expressed vaccine, Flucelvax^11^ and the baculovirus-based HA protein vaccine Flublok^12,15^ in healthy adults. We speculated that vaccines each might have distinct advantages in eliciting CD4 T-cell-dependent antibody responses. The “split” vaccine produced in eggs, which is likely the most impure, has been reported to contain viral RNA that might act as a self-adjuvant^16,17^, while the higher purity of HA in the subunit and recombinant HA could diminish competition among different CD4 T-cell and B-cell specificities. Also, the HA proteins within the vaccines will have N-linked glycosylation patterns that are characteristic of each expression system^18^ and that can affect immunogenicity^19,20^. We have performed side-by-side comparisons of the human immune responses to the influenza vaccines produced using three different platforms. Because of the critical role that CD4 T cells play in protective antibody responses to influenza, here we have focused primarily on quantification of elicitation of CD4 T cells by the vaccines. We have used multiple measures of vaccine-induced CD4 T-cell expansion and activation and include prototypical measures of serum antibody responses such as ELISA and HAI assays. Our studies indicate that, on balance, recombinant purified HA outperforms the virion-based inactivated influenza vaccines in both the cellular and serological responses.

## Results

### An experimental design to evaluate relative efficacy of three distinct influenza vaccine platforms

**Fig. 1:**
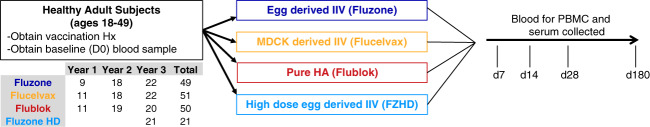
Vaccine comparison study design. Study subjects aged 18–49 years old were randomized into three cohorts, each receiving a licensed seasonal influenza vaccine. The vaccine formulations administered were egg-derived split-vaccine Fluzone quadrivalent (blue), mammalian cell-based Flucelvax (gold), recombinant HA made in the baculovirus system Flublok (red) and egg-derived split-vaccine Fluzone High (HD) dose (light blue). Subjects were enrolled for a single season in one of the vaccine groups. Over the course of 3 consecutive seasons 152 healthy subjects were divided into the first three vaccine groups as indicated in the left-hand portion of the Figure. In the third season, an additional 21 subjects were enrolled in the Fluzone HD group. Blood samples were taken at day 0 (D0) prior to vaccination, and day 7 (D7, for flow cytometry, which is peak for some subsets of CD4 cells at this time), day 14 (D14, for EliSpot assays that peak at this time point) and day 28 (D28, neutralizing antibody) post vaccination. Sera for HAI assays were sampled at day 0 and day 28.

In three successive seasons (2015–2016, 2016–2017 and 2017–2018), subjects were enrolled in a vaccination study to evaluate both their CD4 T-cell and B-cell responses to influenza vaccination. An independent group of subjects in each year was randomized and divided into three cohorts that received one of the three distinct formulations of licensed vaccines, Fluzone^14^, Flucelvax^11,21^ Flublok^22^. The characteristics of the cohorts in each year with regard to age, sex and self-reported vaccine history are shown in Supplementary Table 1. The overall study design is shown in Fig. 1 which shows the schedule of sampling. Sera was sampled at day 0 (prior to vaccination) and day 28, while PBMC were sampled at day 0 and several times post vaccination (day 7, day 14 and day 28 and day 180). The vaccine composition for each season is illustrated in Supplementary Fig. 1. The strains of influenza viruses and HA proteins included in the vaccines differed according to the CDC recommendations and the vaccine manufacturer. One notable feature of the vaccines in this regard is the variable composition of Influenza B in the vaccines, shown in Supplementary Fig. 1. While Fluzone was quadrivalent in all three years, with both lineages of influenza B (reviewed in refs ^23,24^), trivalent vaccines, composed of H1N1, H3N2 and one strain of influenza B, were produced by manufacturers of Flucelvax and Flublok in year 1, after which Flucelvax was quadrivalent in years 2 and 3. Flublok remained trivalent until year 3. Also, Flublok is licensed to be administered at 45 μg of each HA, while Flucelvax and Fluzone are formulated at 15 μg of each HA.

Several assays were performed in order to assess CD4 T-cell responses to vaccination. The major method used to evaluate expansion of CD4 T cells specific for the distinct HA proteins was through peptide-stimulated cytokine EliSpot assays, measured at day 0, prior to vaccination, and at day 14 post vaccination, representing the peak of the response. Peptides used for stimulation of CD4 T cells are overlapping peptide sets corresponding to the entire translated sequence of each HA type (H1, H3 or HA-B), allowing assessment of all potential epitope specificities. Also included was a control pool of peptides from a pathogen that the cohorts in the US would not have naturally been exposed to. Use of this type of control allows a more accurate “background” to be measured with similar peptide quantity and organic solvents^25^.

### Quantification of human HA-specific CD4 T-cell responses to vaccination reveals enhanced efficacy of Flublok, relative to split and subunit influenza vaccines

We first examined total CD4 T-cell expansion to all HA-derived epitopes and quantified these as the gain in influenza HA-specific cytokine producing cells from day 0 to day 14. The rationale for this first global assessment is due to the nature of CD4 T-cell recognition of antigenic peptides. Elicitation of CD4 T cells by influenza vaccines requires host HLA class II molecules to bind and present peptides derived from the H1, H3 and HA-B proteins. HLA is highly variable in humans^26,27^ and thus each subject likely presents a distinct constellation of vaccine HA-derived peptides. Therefore, without any knowledge of the HLA class II molecules expressed, it is not possible to predict the potential of any subject to respond to the influenza-derived epitopes. Accordingly, we thought it valuable to first examine responses in the aggregate, where the cytokine responses for all the HA contained in the vaccines were summed.

**Fig. 2:**
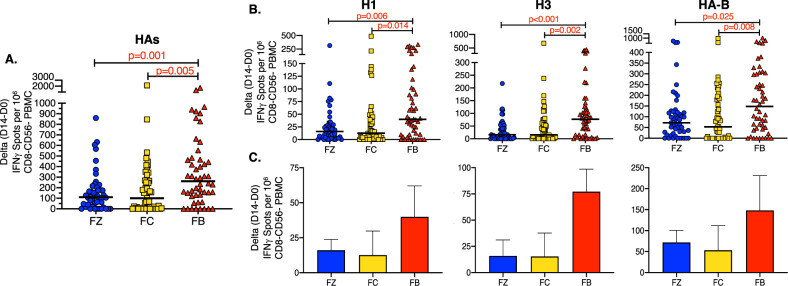
Human HA-specific CD4 T-cell responses post vaccination. CD4-enriched cells from the vaccinated subjects were stimulated with overlapping pools of peptides from H1, H3 and HA-B proteins and evaluated for IFN-γ production using cytokine EliSpot assays. The responses for the 3 groups Fluzone (blue), Flucelvax (gold) or Flublok (red) are indicated. In **a**, the CD4 T cells expanded between day 0 and day 14 for each HA peptide pool tested were summed. The data are represented as the change in the response between day 0 and day 14 (D14–D0) with the median response indicated by a black line. In **b**, the expansion of CD4 T cells for each HA peptide set is shown for the HA protein that is indicated above each panel. H1 is shown on the left, H3 in the middle and HA-B on the right. The top panels illustrate the response of each individual, represented as a single dot with the spread in reactivity of the population and the median value indicated by a black line. The bottom bar graphs indicate the average response of the individuals shown in the top panel and the 95% confidence interval of each group for each HA. The *p* values shown were calculated by the Wilcoxon rank sum test.

These analyses, shown in Fig. 2a, are represented as spots per million of influenza-reactive CD4 T-cell-enriched populations, detected at day 14, with the value quantified at day 0 subtracted. This “delta” value represents the vaccine-induced CD4 T-cell expansion. These data show first, that there is a wide range in responses across individuals within the cohorts and in each different vaccine response, ranging from a total gain of more than 1800 influenza-reactive cells in a few subjects, to very low to undetectable gains in responses for many subjects. Second, comparing across the three vaccine platforms, there is a statistically strong ranking of CD4 T-cell expansion, with Flublok eliciting the most HA-specific CD4 T cells relative to Flucelvax (*p* = 0.005) and Fluzone (*p* = 0.001).

The CD4 T-cell responses gained from day 0 to day 14, quantified for individual HA proteins contained in the vaccines, are shown in Fig. 2b, where reactivity to H1 (left panel), H3 (middle panel) and HA-B (right panel) are presented. For HA-B, the peptide set derived from B/Florida/04/2006 was used, because the two lineages of HA-B have >90% sequence identity and thus the vast majority of antigenic peptides from the two lineages are shared. For each HA, variability among individuals is shown in the top panel and the average values with the 95% confidence interval are shown in the bottom panel. As was observed in the aggregate HA-summed data, Flublok vaccination led to the most robust gain in CD4 T cells, and was observed for all HA types. Increases in circulating CD4 T cells elicited by Flublok for H1 epitopes were significantly greater than Flucelvax (*p* = 0.014) and Fluzone (*p* = 0.006). The same pattern was observed for CD4 T cells specific for H3 epitopes induced by Flublok vaccination relative to Flucelvax (*p* = 0.002) and Fluzone (*p* < 0.0001). The hierarchy of responses for CD4 T-cell reactivity to the influenza HA-B protein was less striking among the vaccines, although still statistically significant. The lower HA-B specific CD4 T-cell responses elicited by Flucelvax and Flublok may be due to lower quantities of HA-B in trivalent vaccines year 1, and years 1 and 2, respectively, relative to the quadrivalent Fluzone vaccine for each of the three seasons. In general, the data on CD4 T-cell expansion is consistent with a higher immunogenicity of Flublok over Flucelvax and Fluzone in elicitation of CD4 T cells.

**Fig. 3:**
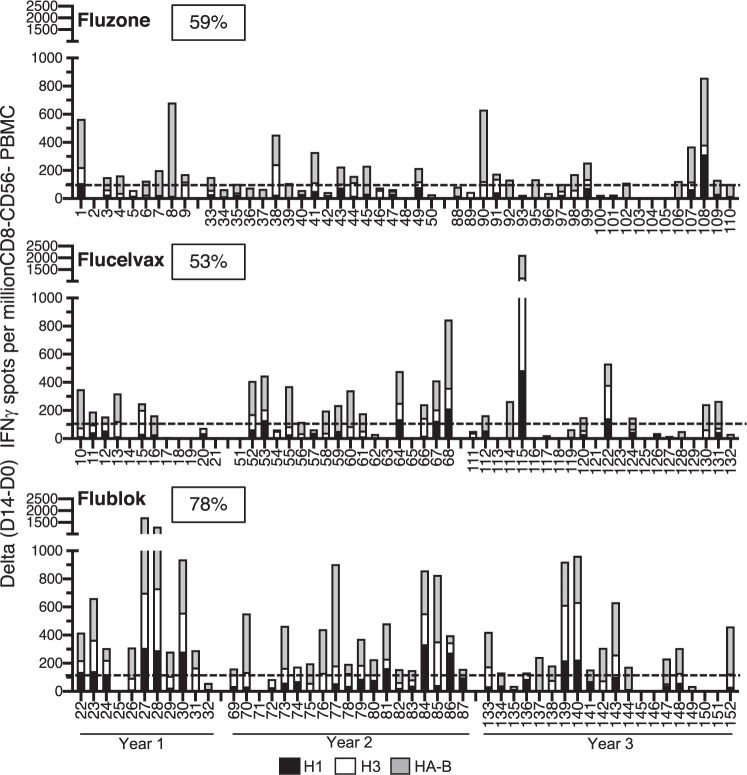
HA-specific CD4 T-cell responses post vaccination for individuals. CD4-enriched populations of cells from the subjects vaccinated with Fluzone (top), Flucelvax (middle) or Flublok (bottom) were stimulated with pools of peptides from the HA indicated. H1-specific responses are shown in black bars, H3 in white and HA-B in grey. IFN-γ cytokine producing cells were quantified using EliSpot assays. The data are represented as a stacked bar for each vaccinated individual and the year of the study is indicated at the bottom of the graphs. A dotted line indicates the 100-spot cutoff used to estimate a positive CD4 T-cell response. The boxed number at the top of each panel is the percent response-based frequency using this cutoff for each vaccine.

We next sought to gain insight into the HA-specific responses in CD4 T-cell expansion among individuals and whether the patterns were similar across the three seasons of this study. In order to evaluate these issues, the cytokine EliSpot data, again represented as the difference in HA-specific CD4 T cells detected at day 14 relative to day 0, was plotted for each individual as stacked bar graphs for each HA response (H1, H3 and HA-B), as shown in Fig. 3. Reactivity to H1 is shown in black bars, with H3 in white and HA-B shown as grey. Fluzone vaccine responses are shown in the top panel, Flucelvax in the middle and Flublok shown in the bottom panel. These analyses illustrate the pattern of responses, subject by subject (indicated as a subject number beneath each graph) over the course of the 3-year study. Several striking observations were made when the data were analyzed in this fashion. The first and most striking difference among the subjects is the tremendous variability in the specificity of the CD4 T-cell response pattern. Some subjects display a balanced response to all three HA proteins, while others show a dominant response to one HA protein, most commonly to HA-B, a pattern particularly prominent in the responses to Fluzone. Many, but not all, subjects that exhibit an extremely high response to one HA also display a robust CD4 T-cell response other HA proteins (Supplementary Fig. 2). The relative proportion of their response dedicated to each separate HA varied from a roughly equal distribution (for example, by subject 30 in the Flublok group and subject 115 in the Flucelvax group), to an almost exclusive reactivity to one HA, as is observed in the response in subject 8 in the Fluzone group and subjects 114 and 137 in the Flucelvax and Flublok groups, respectively, who showed exclusive gains in reactivity to HA-B. We do not know where this variation in CD4 T-cell dominance toward one HA over others derives from, but it is likely in part due to the previously discussed HLA-dependent presentation of peptide epitopes, in some subjects selecting primarily for H1-derived peptides and in others, H3 or HA-B. Although it is possible that biases in reactivity to different strains of HA relate to immune imprinting by infection (reviewed in refs ^28–30^), the preference does not link to date of birth (Supplementary Fig. 3), perhaps because of the small sampling size and somewhat narrow age range of our subjects (18–49 years old). However, strain biases in CD4 T cells could be due to overall abundance of memory cells that were established from or boosted by infections which are often subclinical in nature and not recorded by physicians or the subjects themselves. In order to gauge relative responses in a different way, a cutoff of a gain of 100 cytokine-producing cells was set as a “positive response” between day 0 and day 14, indicated as a dotted line in each panel. The percentage of responders are shown in the box within each panel for the vaccine used. By this measure, 78% of the subjects vaccinated with Flublok demonstrated a CD4 T-cell response, while Flucelvax and Fluzone averaged 53% and 59%, respectively. Thus, by both the overall total expansion of CD4 T cells and the percent responders, Flublok exhibits the highest vaccine efficacy.

**Fig. 4:**
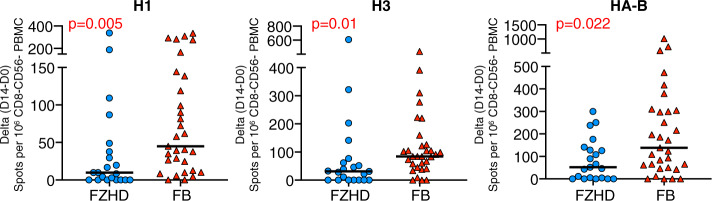
The more robust CD4 T-cell response to Flublok post vaccination is not determined by HA dose within the vaccine. A group of 21 healthy subjects, aged 18–49 years old were vaccinated with high dose trivalent Fluzone (blue) and compared to those subjects in years 1 and 2 that received trivalent Flublok (red). CD4-enriched populations were stimulated with pools of peptides from H1, H3 and HA-B and evaluated for IFN-γ production using cytokine EliSpot assays. The data are represented as the change in the response between day 0 and day 14 (D14–D0), with the median response indicated by a black line. The specific HA reactivity is indicated above the panels, with H1 on the left, H3 in the middle and HA-B on the right. The *p* values shown were calculated by the Wilcoxon rank sum test.

One question that can be raised about the overall superior performance of Flublok in eliciting CD4 T cells after vaccination, relative to Fluzone and Flucelvax, was whether the increased quantities of HA accounted for its greater efficacy. As discussed, Flublok is licensed at 45 μg of each HA (H1, H3 and HA-B) per dose, while standard split and subunit influenza vaccines are delivered at 15 μg of each HA per dose^31^. To address whether the disparities in immunogenicity were due exclusively to this higher HA content, in year 3, we enrolled an additional cohort of subjects to be vaccinated with Fluzone high dose, “FZ-HD”, that is formulated at 60 μg of each HA per dose. This vaccine was developed primarily for elderly subjects who tend to have more modest responses to influenza vaccination^32–36^. However, for the issue addressed here, all subjects were of the same age range (18–49 years old). The FZ-HD is formulated as a trivalent influenza vaccine, with only one lineage of HA-B. To best address this issue of higher HA content, we analyzed the CD4 T-cell responses of FZ-HD and the responses to trivalent Flublok, collected in years 1 and 2, where the HA-B content would be most similar, in parallel. CD4 T-cell expansion (day 14-day 0) of trivalent Flublok and trivalent FZ-HD were compared and gains in CD4 T-cell reactivity to H1, H3 and HA-B were quantified in Fig. 4. Despite being 25% lower in dose (45 μg for Flublok vs. 60 μg for FZ-HD), Flublok still elicited greater CD4 T-cell expansion than Fluzone (*p* = 0.02–0.005). Interestingly, when compared side by side, the responses of subjects to Fluzone and Fluzone HD were not statistically different in this age group (Supplementary Fig. 4), suggesting that the positive impact of dose may be primarily exhibited by the elderly. We conclude from this comparison that there are qualitative advantages to the Flublok vaccine in CD4 T-cell immunogenicity relative to the vaccines made from extracts of virions.

**Fig. 5:**
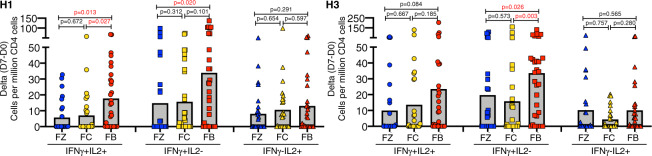
Polyfunctional CD4 T-cell response to vaccination with three vaccine platforms. PBMC from subjects vaccinated with Fluzone (blue), Flucelvax (gold) or Flublok (red) were stimulated in vitro with pools of peptides derived from H1 (left) or H3 (right) for 16 h. Brefeldin A and monensin were added for the last 8 h of culture to block cytokine secretion. H1- or H3-specific CD4 T cells were identified based on expression of CD69 + and the intracellular cytokines IL-2 and IFN-γ. Shown is quantification of cells producing both cytokines, IFN-γ + IL2 + (left), IFN-γ + IL2- (middle) and IFN-γ-IL2 + (right), using the gating scheme shown in Supplementary Fig. 5. The data are represented as the gain in the number in antigen-reactive, cytokine-positive cells between day 0 and day 7. The average response is indicated by the grey bar and the individual responses are indicated by the scatter. The *p* values shown were calculated by the Wilcoxon rank sum test.

To determine if assessment of multiple cytokines would reveal different patterns of CD4 T-cell recruitment among the three vaccines, intracellular cytokine staining assays (ICS) were employed. Unpublished studies by our laboratory indicated that IL-2 and IFN-γ production were the most readily quantified for influenza-specific CD4 T cells post vaccination. PBMC samples were stimulated with peptides derived from H1 or H3 proteins and cytokines were captured after a secretion block^37,38^. HA-B reactivity was not included here because the samples from years 1 and 2 of these studies contain different quantities of total HA-B (e.g. quadrivalent vs. trivalent) in the vaccines and thus a direct comparison across the vaccines was not possible. The results of the ICS assays are shown in Fig. 5. Cells sampled at day 7, representing early emerging responding cells, producing both cytokines (IFNγ + IL2 + ) are shown in the first panel, while cells producing only IFN-γ (IFNγ + IL2-) or only IL-2 (IFNγ-IL-2 + ) are shown in the middle and left panels, respectively. These experiments revealed that, in general, the patterns of cytokine production elicited by the three vaccines were similar and that the major subset of expanded cells produced IFN-γ alone. This finding is in keeping with the general Th1 phenotype of CD4 T cells responding to influenza infection^39–41^. By these assays, Flublok appears to elicit the most CD4 T cells specific for H1 in both (IFNγ + IL2+) double-producers and cells exclusively producing IFN-γ. The same trend was found for H3 reactivity.

### Flublok outperforms Fluzone and Flucelvax in eliciting influenza HA-specific antibody responses

**Fig. 6:**
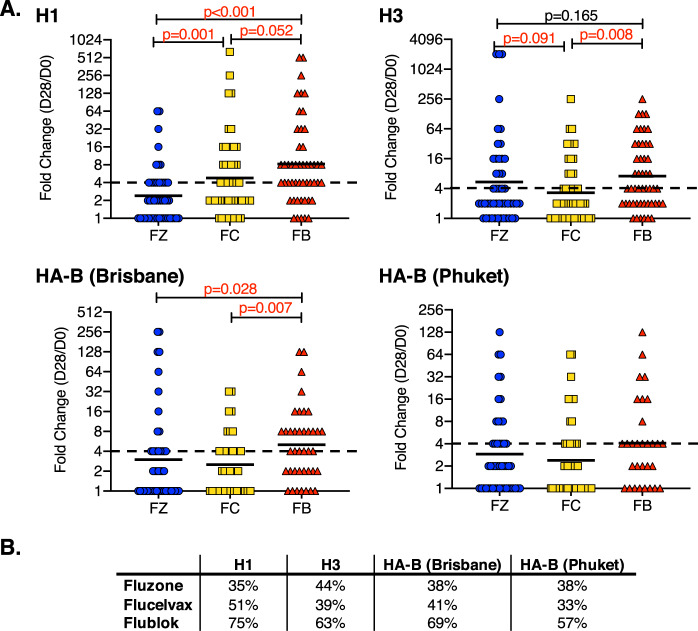
Antibody responses to vaccination as measured by HAI assay. The neutralizing serum antibody response at day 0 before vaccination and day 28 post vaccination was measured by HAI assays from subjects vaccinated with Fluzone (blue), Flucelvax (gold) or Flublok (red). Shown in **a** is the fold-change between day 0 and day 28 (D28/D0) for each of the HA strains contained in the vaccine, H1 (top left), H3 (top right), HA-B Brisbane (Victoria lineage, bottom left) and HA-B Phuket (Yamagata lineage, bottom right). The data from individuals are represented by the scatter and the geometric mean is indicated by a black line. A dotted line indicating the fourfold response, typically used to indicate a positive antibody response, is shown. The *p* values were calculated by the Wilcoxon rank sum test. The table in **b** indicates the percent responders for each vaccine, indicated on the rows to the left, and each HA, indicated on the columns above, based on a fourfold or greater HAI response.

As part of these comparative analyses of vaccine responses, we performed standard HAI assays on the serum collected at day 0 (pre-vaccination) and day 28 (post vaccination). This assay is widely used as a correlate of protective immunity^42,43^. The results, shown in Fig. 6a, are represented as the traditional “fold-change” from day 0 to day 28. The data shown in Fig. 6a illustrates the range in responses among individual subjects. The HAI responses were quite variable among the subjects in each of the vaccine groups, in agreement with the results of CD4 T-cell studies. Although many subjects displayed less than a fourfold change in HAI titers, some of the vaccinees exhibited more than a 50-fold change in HAI titers after vaccination. For H1N1 A/California virus, the Flublok outperformed both the Fluzone and the Flucelvax, with the Flucelvax showing an intermediate efficacy. For H3N2-specific responses, each of the three vaccines elicited responses that were more comparable to each other, with Flublok showing a statistically greater response than Flucelvax. Because Flublok and Flucelvax were only formulated as trivalent vaccines in some seasons, only the matched HAI Influenza B data were analyzed. Flublok elicited significantly greater influenza B (Brisbane-specific) HAI antibodies than Flucelvax and Flublok. Although the same trend was seen with responses to influenza B Phuket, the differences between the different vaccine groups did not reach statistical significance. When percent responders were calculated based on whether their HAI titers exhibited at least a fourfold change (Fig. 6b), Flublok-vaccinated subjects elicited the greatest number of responders to H1N1, H3N2, Influenza B Brisbane and Phuket (75%, 63%, 69% and 57%, respectively).

**Fig. 7:**
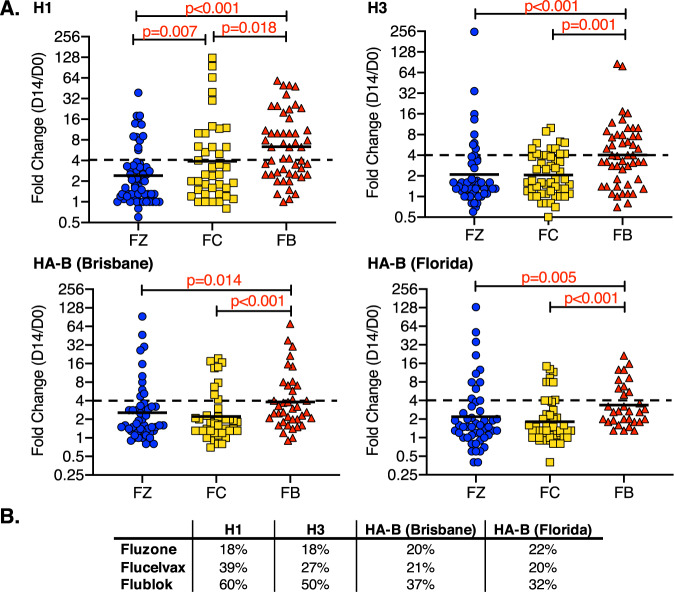
Total HA-specific antibody response quantified by ELISA. Total HA-specific antibody responses of subjects vaccinated with Fluzone (blue), Flucelvax (gold) or Flublok (red) are shown. Anti-HA antibody levels collected before vaccination and day 14 post vaccination were measured by HA-specific IgG ELISA assays. The relative titers were quantified based on the dilution of antibodies required to reach a fixed OD405 signal on the linear portion of the antibody titration curve. Shown in panel **a** is the fold-change between day 0 and day 14 (D14/D0) for each of the HA strains contained in the vaccine, H1 (top left), H3 (top right), HA-B Brisbane (Victoria lineage, bottom left) and HA-B Florida (a surrogate for B/Phuket, Yamagata lineage, bottom right). Responses among individuals are represented by the scatter and the geometric mean is indicated by a black line. A dotted line indicates the fourfold response. The *p* values shown were calculated by the Wilcoxon rank sum test. In **b**, the table below, indicates the percent responders for each vaccine. The vaccine is indicated to the left and the specific HA protein on the columns above, based on a fourfold or greater response.

It is now well accepted that many antibodies to HA have protective function in infections, including mechanisms that include antibody-dependent cellular cytotoxicity (ADCC) and antibody-dependent cellular phagocytosis (ADCP) (reviewed in refs ^44–46^). These are conveyed by both HA head- and HA stalk-specific antibodies. We therefore sought to measure gains in HA-specific antibodies that were not dependent on ability to neutralize influenza virus. Accordingly, all H1, H3 and HA-B-specific serum antibodies in vaccine subjects were quantified using IgG ELISAs and the fold-change from day 0 to day 14 was calculated for each antigen. Figure 7 shows the results of these assays. Figure 7a illustrates that for each influenza antigen, Flublok serum antibody responses exhibited the greatest fold-change between day 0 and day 14. As in the other assays, there was substantial variance in response magnitude among subjects, with some subjects exhibiting more than a 100-fold gain in total HA-specific antibodies at day 14. Despite this range in responsiveness displayed in each cohort, statistical treatment of the data revealed strikingly significant differences. For H1, H3, and HA-B, Flublok performed greater than either of the other two vaccines, with *p* values often <0.005, relative to Flucelvax and Fluzone. The precise statistics depend on the HA antigen tested. For H3 and HA-B, the responses elicited by Flucelvax and Fluzone were generally comparable. When percent responders were calculated based on whether their relative titers exhibited at least a fourfold change (Fig. 7b), Flublok-vaccinated subjects elicited the greatest number of responders for all viral strains.

## Discussion

In the study reported here, we focused exclusively on the comparative efficacy of three licensed influenza vaccines that are made on different platforms and that differ in viral protein composition. These studies revealed that for each HA protein in the vaccine, Flublok elicited the most robust and statistically significant CD4 T-cell responses, relative to Fluzone and Flucelvax, whether calculated as the mean response for each subject or as the fraction of responders. In agreement with the measures of CD4 T-cell responses, quantification of serum antibody production using ELISA and HAI assays showed the same hierarchy of efficacy. The disparity observed across the three vaccine platforms was greatest between Fluzone and Flublok, with Flucelvax most commonly intermediate between the two other vaccines.

The mechanisms underlying the greater responses induced by the Flublok vaccine are not yet clear. It seems likely that one contributor is the higher quantity of HA antigen administered per dose. However, our data show that even when a split influenza vaccine is administered at 4X the normal dose (Fluzone HD), the split vaccine still performed less well than did Flublok in eliciting CD4 T cells. This suggests that beyond quantity, qualitative features of the influenza vaccines differ. One source of variability among the vaccines is the distinct N-linked glycosylation patterns that the HA proteins are likely to display. Biochemistry studies have shown that the SF9 expression system yields paucimannose carborhydrates and high mannose on HA, while mammalian and egg-based expression systems produce HAs with high mannose, complex and hybrid structures^47^ whose ratios depend on the HA under study^18^. Such differences can affect antigen handling and recognition by C-lectin receptors expressed on antigen presenting cells (APC) such as DC-SIGN and DEC-205 (reviewed in ref. ^48^). In fact, studies by De Vries et al. showed that in animal models, HA proteins with terminal mannose, enriched on insect cell derived HA, were less immunogenic^19^. The antigen handling of influenza vaccines after human intramuscular vaccination, and the participating APC, are not well defined but can be envisioned to be affected by the variable N-linked sugars on the HA produced in the different vaccine platforms.

An additional explanation for our findings is that the complex viral protein composition of vaccines made from extracts of influenza virions in some way diminishes the CD4 T-cell and B-cell reactivity to the HA components in the vaccine. We have recently shown that split influenza vaccines such as Fluzone, contain abundant M1 and NA as well as NP protein^49^ in agreement with the studies of others^50–52^. The consequences of this in the response are interesting to consider. Due to repeated confrontation by humans to influenza via vaccination and infection, most individuals have circulating memory CD4 T cells^25,53–57^, B cells^58,59^ and antibodies to these components^60–62^. This mixture of influenza proteins may elicit potentially antagonistic or competitive T-cell and B-cell responses with HA. Also, circulating antibodies may disproportionately impact vaccines made of complex mixtures of viral proteins. Studies by many groups (reviewed in refs ^63,64^) have shown that individuals who were vaccinated in previous seasons have diminished HA-specific responses to influenza vaccination. We have found that the lower antibody responses in previously vaccinated subjects also exhibit diminished CD4 T-cell responses^65^. Others have shown that pre-existing serum antibody levels are also associated with lower subsequent B cell and antibody responses to the influenza vaccines^66,67^. Residual antibodies in repeatedly vaccinated subjects might block or clear some of the administered vaccines. Our most recent studies show that different viral proteins in licensed vaccines can form heterologous complexes, such that HA can interact with M1 and NA proteins within the vaccines^49^. These complexes may be cross-cleared by pre-existing antibodies to any of the viral components that interact with HA. Fluzone, being the most complex and lower in initial dose than Flublok, and also enriched in these other viral proteins may suffer the most from the effects of circulating antibodies and competition during the responses to influenza vaccination. Testing of this model, as well as the relationship between CD4 T-cell response specificity and magnitude with B-cell responses will be challenging due to our limited understanding of antigen handling in vivo after vaccination and the multiple subsets of human CD4 T cells that vary in their ability to be recruited in the response and to potentiate B-cell responses. However, these issues are important to consider in any proposed vaccine designed to protect from complex viral pathogens with a dominant protective antigen such as the HA protein of influenza viruses.

There are some limitations of our studies and parameters measured in the vaccine response that we have not evaluated here. First, we have not yet quantified whether the different influenza vaccines “seeded” more memory influenza-specific cells for long-term immunity. In the short time of our sampling, (up to day 180 post vaccination), there was only modest and variable changes in antibody and CD4 T-cell responses that were equivalent across the vaccines. Also here, we have not yet fully characterized some of the potentially important features of influenza-specific CD4 T cells, such as the specificity of the Tfh that emerge at day 7, important in the antibody response, or the full phenotypic characteristics of the expanded cells, such as expression of memory markers, homing markers and chemokine receptors. Also, it is important to note that in contrast to some of the newer vaccine strategies aimed at generating broadly protective immunity to influenza^68,69^, the vaccines tested here are designed to elicit protective antibodies, and the abundance and phenotype of CD4 T cells elicited in responses to vaccines specifically to engage T cells will be critical to study. We also only sampled CD4 T cells that emerge into peripheral blood after vaccination and not those CD4 T cells that may persist in the germinal centre after vaccination, now being sampled by some investigators^70^. All of these parameters could be relevant to judging vaccine efficacy and long-term immunity and are the subject of current and future studies by our group as well those of others.

## Materials and methods

### Influenza vaccine study groups and sampling

After approval by the Division of Microbiology and Infectious Diseases (DMID) and the UR Research Subjects Review Board (protocol l5-0055), subjects who provided informed consent were enrolled to evaluate both their CD4 T-cell and B-cell responses to one of three licensed influenza vaccine (Illustrated in Fig. 1). Self-reported vaccine histories of each subject indicated that a similar number in each vaccine group (approximately 60%) had been vaccinated in the previous season (Supplementary Table 1). In each of 3 seasons, independent subjects were randomized, and vaccinated with either Fluzone, Flucelvax, or Flublok. Blood was obtained on day 0 and days 7, 14, 28 and 180 after vaccination. In year 3, an additional vaccine type, Fluzone High Dose (FZ-HD), was added, and 21 healthy adults in the same age range as the main study were enrolled in this group. Blood specimens were obtained, plasma was removed, and PBMC were isolated and frozen until use.

### Synthetic peptides

Peptide libraries were obtained from BEI Research Repository, NIAID, NIH and included A/California/04/09(H1N1) HA (pH1), A/New York/384/2005(H3N2) HA (H3), and B/Florida/04/2006 HA (HA-B). The peptide pools have >92% sequence identity across the strains included and Influenza B lineages in the vaccines, allowing the vast majority of HA reactive T cells to be quantified. A negative pool of peptides from Sin Nombre virus glycoprotein precursor was used to estimate and subtract background. All peptides from a given protein were combined, with each peptide at a final concentration of 1 μM.

### Enzyme-linked cytokine immunospot assay

T-cell enzyme-linked cytokine immunospot (EliSpot) assays were performed as previously described^25^. In brief, PBMC were thawed, rested overnight in culture, washed and depleted of CD8^+^ and CD56^+^ T cells, using MACS microbeads (Miltenyi Biotec). CD4 T cells typically comprise 50–60% of the enriched PBMC population. CD4-enriched PBMCs were cultured with peptide pools on plates coated with anti-human interferon (IFN-γ) (clone 1-D1K, MabTech, catalogue number 3420-3-1000, 10μg/ml) for 36 h, washed and incubated with biotinylated anti-human IFN-γ (clone 7-B6-1, MabTech, catalogue number 3420-6-1000, 1μg/ml) for 1 h, washed and incubated with alkaline phosphatase-strepavidin (Jackson Laboratories, 1:1000 dilution) for 30 min, and then washed and developed for detection of cytokine producing cells using Vector Blue Substrate kit (Vector Labs), and scored using an Immunospot reader (series 5.2), using Immunospot software, v5.1.

### Intracellular cytokine staining (ICS)

PBMC from pre-vaccination (day 0) and day 7 post vaccination were thawed, rested in culture overnight, washed, depleted of CD69+ cells using MACS microbeads (Miltenyi Biotec), to eliminate cells expressing the activation marker, and stimulated with the peptide pools for 16 h. In the 200 μl culture, 2 μl of Brefeldin A and 4 μl of Monensin (1:100) were added for the last 8 h of stimulation (BD Biosciences, catalogue numbers 555029 and 554724, respectively). Cells were washed and stained for 30 min with Fixable Live/Dead Aqua (Fisher Scientific, catalogue number L34957). Surface and intracellular staining was performed as previously described^71,72^. Antibodies included CD8 (RPA-T8, 1:32 dilution, BD catalogue number 560662), CD4 (RPA-T4, 1:8 dilution, BD catalogue number 555346), CD3 (SK7, 1:8 dilution, BD catalogue number 560176), CD45RA (HI100, 1:8 dilution, catalogue number 563733), IL-2 (MQ1-17H1, 1:4 dilution, catalogue number 564165), IFN-γ (B27, 1:4 dilution, BD catalogue number 562988), CD69 (FN50, 1:4 dilution, BD catalogue number 555532) (BD Biosciences) and CD19 (HIB19) (1:8 dilution, Biolegend catalogue number 302236), with cell fixation and permeabilization performed using the eBioscience FoxP3 cytofix/perm kit. Fluorochrome-stained UltraComp e-Beads (Invitrogen) or ArC Amine Reactive Compensation Beads (ThermoFisher Scientific) were used for compensation. Data were acquired using a BD LSR-II instrument, configured with 488, 633, 407 and 532-nm lasers using FACS DIVA software (BD Bioscience). Sequential gating was performed using FlowJo version 10 (Tree Star). Activated cells of interest were defined as CD69 + CD4 + T cells (CD3+, CD8−) that were positive for cytokine staining (IFN-γ and/or IL-2). The gating scheme is shown in Supplementary Fig. 5.

### Hemagglutination Inhibition Assay

Hemagglutination inhibition assay (HAI) testing was performed as previously described^73^. Sera were treated with receptor-destroying enzyme (Denka Seiken, Tokyo, Japan) and heat inactivated (56 °C, 30 min) prior to use. The egg-derived virus strains used in this study included H1N1 A/California/07/09, H3N2 strains A/Switzerland/9715293/13 and A/Hong Kong/4801/14, and Influenza B strains B/Phuket/3073/13 and B/Brisbane/60/08, obtained from the Influenza Research Repository (IRR). The antibody titers were defined as the reciprocal of the highest dilution that inhibited hemagglutination.

### Enzyme-linked immunosorbent assay

Plates were coated with 200 ng/well purified HA proteins from A/California/04/09 (H1), A/Switzerland/9715293/13 (H3), and HA-B strains, B/Florida/04/06 (a surrogate for B/Phuket), and B/Brisbane/60/08 (obtained from BEIR), rinsed, and blocked with 3% bovine serum albumin in phosphate-buffered saline. Plasma samples, starting at a dilution of 1:50, were added, incubated for 2–3 h at room temperature, washed and incubated sequentially with 100 μL/well alkaline phosphatase-conjugated anti-human immunoglobulin G (1:1000 dilution, MT78, MabTech catalogue number 3850-9 A) and 1 *p*-nitrophenyl phosphate substrate, and scored at 405 nm. The relative titer was determined at a fixed OD, based on the 50% point of the linear portion of the curve.

### Statistics

Data are presented as the frequency of cytokine-producing cells per million CD8- and CD56-depleted PBMCs for EliSpot assays, the number of cells per million CD4 cells for flow cytometry, or for plasma and sera, fold-change of relative titers from day 0. Where the delta was negative, the value was set to zero. Statistical differences between vaccine groups were calculated as *p* values using the nonparametric Wilcoxon Rank sum test. The findings hold after multiple test adjustments to control for type I error. There were no differences in age and previous vaccination status among the three vaccine groups (Supplementary Table 1), and thus these variables were not included in analysis.

### Reporting summary

Further information on research design is available in the Nature Research Reporting Summary linked to this article.

## Supplementary information

Supplementary Information

Reporting Summary

## Data Availability

The full complement of data accumulated for these studies is available upon request.
