# Supplementary material for: Recombinant HA-based vaccine outperforms split and subunit vaccines in elicitation of influenza-specific CD4 T cells and CD4 T cell-dependent antibody responses in humans
Source: NPJ Vaccines. 2020 Aug 26;5:77. doi: 10.1038/s41541-020-00227-x (PMC7450042; doi:10.1038/s41541-020-00227-x)
Supplement: Supplementary file 1 — Supplementary Information [file 41541_2020_227_MOESM1_ESM.pdf]

**2015** (Yr1)	**Fluzone** (15μg ea HA)	**Flucelvax** (15μg ea HA)	**Flublok** (45μg ea HA)
H1N1	A/California/07/2009	A/Brisbane/10/2010	A/California/07/2009
H3N2	A/Switzerland/9715293/2013	A/South Australia/55/2014	A/Switzerland/9715293/2013
B/Victoria	B/Brisbane/60/2008		
B/Yamagata	B/Phuket/3073/2013	B/Utah/9/2014	B/Phuket/3073/2013

**2016** (Yr2)	**Fluzone** (15μg ea HA)	**Flucelvax** (15μg ea HA)	**Flublok** (45μg ea HA)
H1N1	A/California/07/2009	A/Brisbane/10/2010	A/California/07/2009
H3N2	A/Hong Kong/4801/2014	A/Hong Kong/4801/2014	A/Hong Kong/4801/2014
B/Victoria	B/Brisbane/60/2008	B/Hong Kong/259/2010	B/Brisbane/60/2008
B/Yamagata	B/Phuket/3073/2013	B/Utah/9/2014	

**2017** (Yr3)	**Fluzone** (15μg ea HA)	**Flucelvax** (15μg ea HA)	**Flublok** (45μg ea HA)
H1N1	A/Michigan/45/2015 X-275	A/Singapore/GP1908/2015 IVR-180	A/Michigan/45/2015
H3N2	A/Hong Kong/4801/2014 X-263B	A/Singapore/GP2050/2015	A/Hong Kong/4801/2014
B/Victoria	B/Brisbane/60/2008	B/Hong Kong/259/2010	B/Brisbane/60/2008
B/Yamagata	B/Phuket/3073/2013	B/Utah/9/2014	B/Phuket/2073/2013

Supplementary Figure 1. Vaccine Composition. The viral strains included in each of the licensed vaccines used in this study.

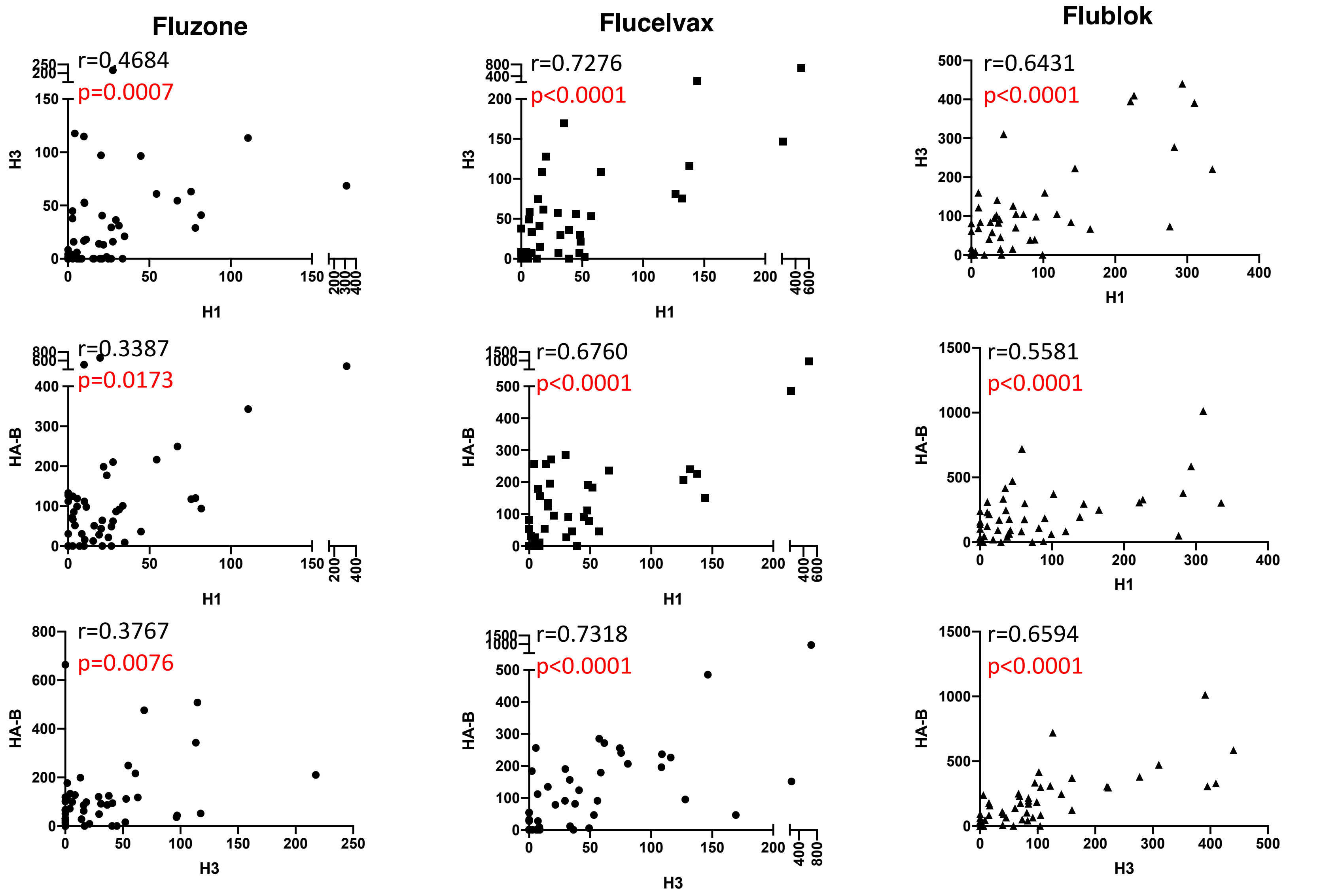

Supplementary Figure 2. Relationships between HA specific CD4 T cell responses post vaccination. Human subjects were vaccinated with one of the three licensed influenza vaccines Fluzone (left), Flucelvax (middle) or Flublok (right). CD4-enriched populations were stimulated with pools of peptides from the HA indicated on each axis. IFNγ cytokine producing cells were quantified using EliSpot assays. The data is presents as the change between day 14 and day 0 (D14-D0) of the number of IFNγ producing cells per million CD8-CD56- PBMC. The correlation between the CD4 responses for H1 and H3 (top row), H1 and HA-B (middle row) and H3 and HA-B (bottom row) are shown. The r and p values are indicated in the top left corner of each panel and were calculated using the non-parametric Spearman correlation.

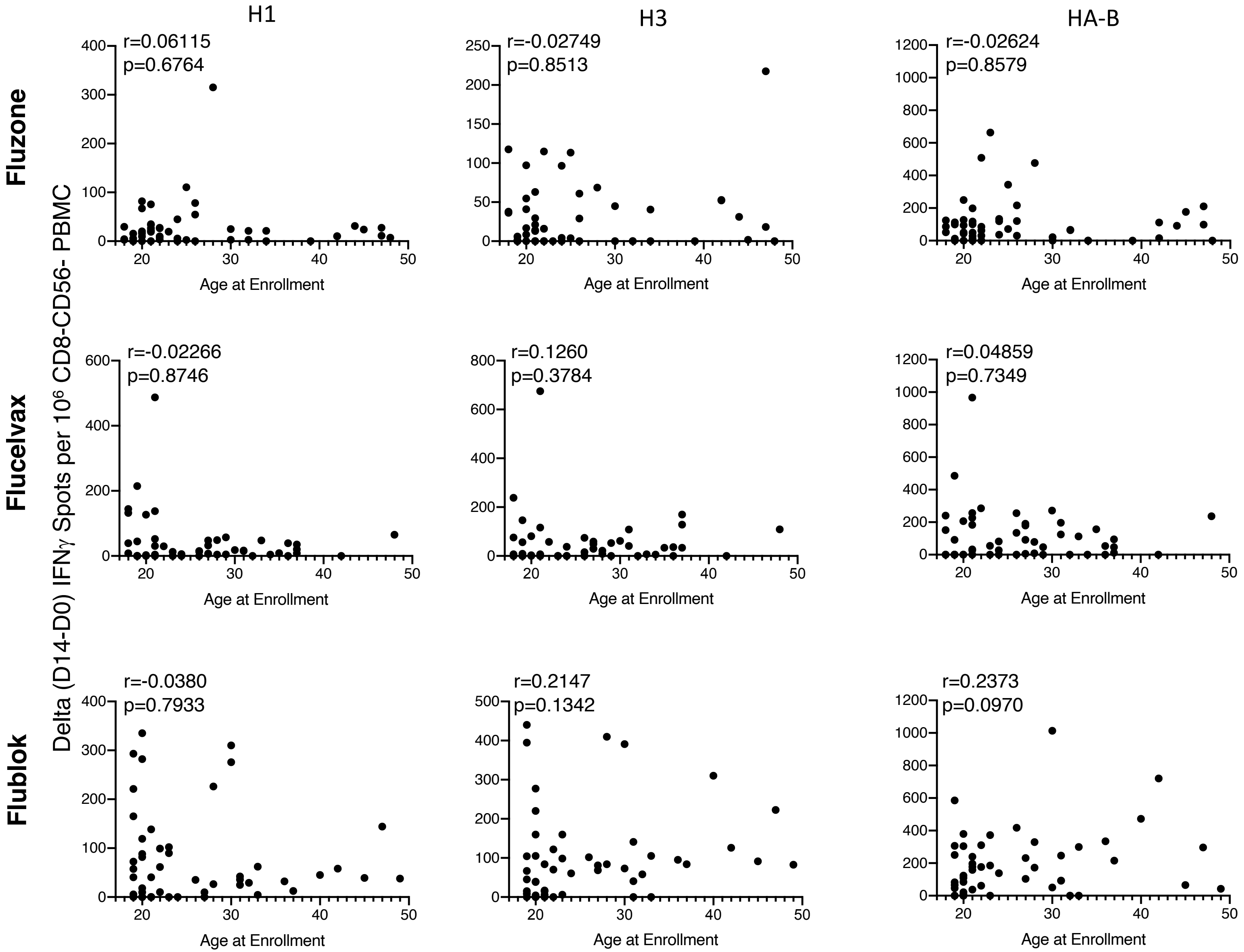

Supplementary Figure 3. The pattern of CD4 T cell responses post vaccination based on age. Human subjects were vaccinated with one of the three licensed influenza vaccines, Fluzone (top), Flucelvax (middle) or Flublok (bottom). CD4-enriched populations were stimulated with the pools of peptides from each HA protein indicated above each column. IFN-γ cytokine-producing cells were quantified using EliSpot assays. The data is presented as the change in response between day 14 and day 0 (D14-D0) of the number of IFN-γ producing cells per million CD8-CD56- PBMC, indicated on the y-axis and age is indicated on the x-axis. The r and p values are indicated in the top left corner of each panel and were calculated using the non-parametric Spearman correlation.

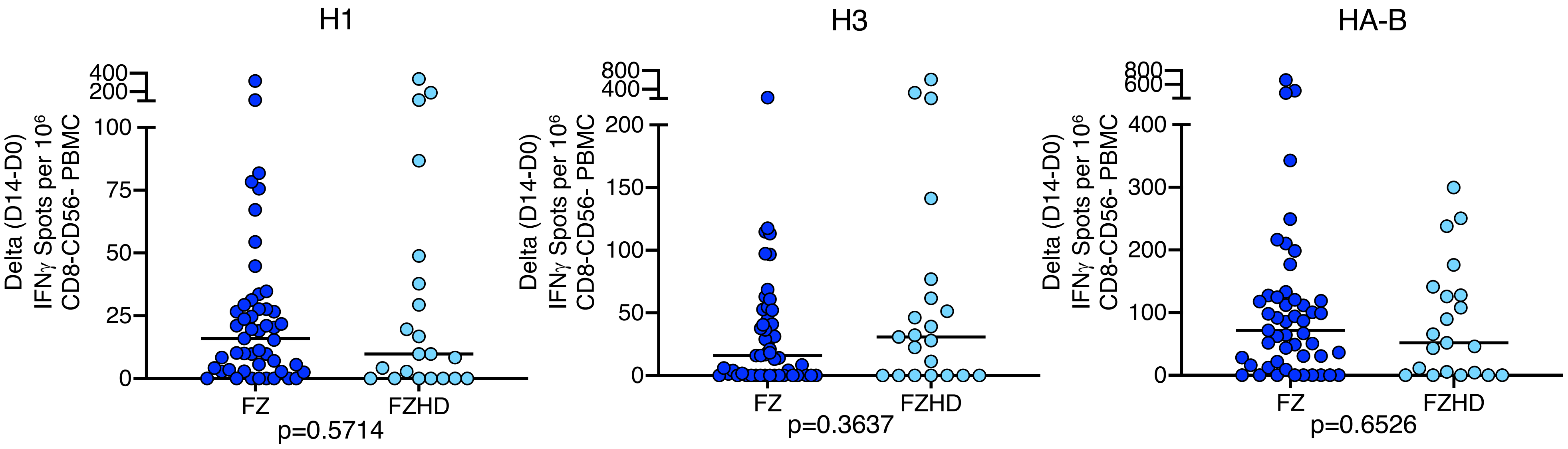

Supplementary Figure 4. CD4 T cell response post vaccination is not determined by HA dose within the vaccine. A group of 21 healthy subjects, aged 18-49 years old were vaccinated with high dose trivalent Fluzone (light blue) and compared to subjects that received quadrivalent Fluzone (dark blue). CD4-enriched populations from each group were stimulated with pools of peptides from H1, H3 and HA-B and evaluated for IFN-γ production using cytokine EliSpot assays. The data are represented as the change in the response between day 0 and day 14 (D14-D0) with the median response indicated by a black line. The specific HA reactivity is indicated above the panels, with H1 on the left, H3 in the middle and HA-B on the right. The p values shown were calculated by the Wilcoxon rank sum test.

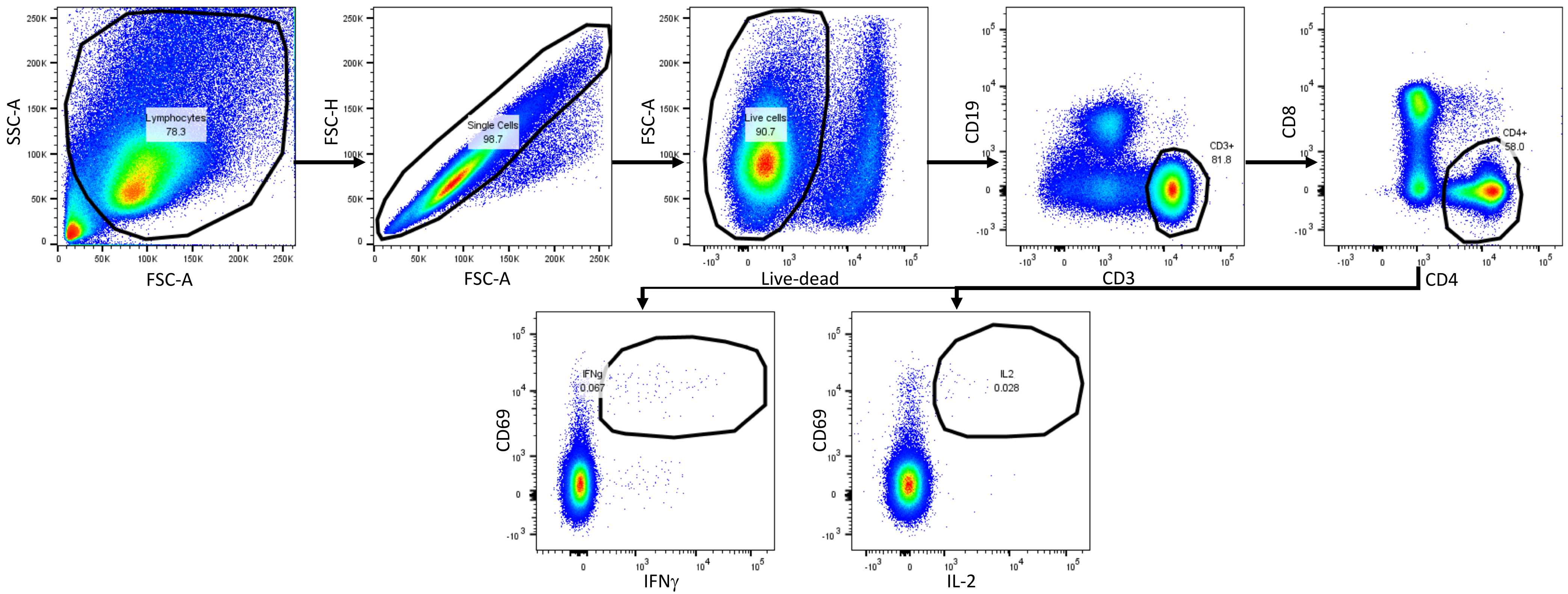

Supplementary Figure 5: Representative plot of the sequential gating strategy utilized to identify activated cells that express IFNγ and/or IL-2. PBMCs isolated from vaccinated subjects were stimulated in vitro with peptide pools of interest, with brefeldin A and monensin added for the last 8 hours of culture to block cytokine secretion. Surface and intracellular cytokine staining were performed and data acquired on a BD LSR-II flow cytometer. After exclusion of debris and doublets, cells were gated on live, CD3+, CD4+ T cells that expressed CD69 together with IFNγ and/or IL-2.

Fluzone				Flucelvax				Flublok			
Subject No.	Gender	Previous Vaccination	Age	Subject No.	Gender	Previous Vaccination	Age	Subject No.	Gender	Previous Vaccination	Age
1	M	N	25	10	M	N	30	22	F	Y	21
2	F	Y	48	11	F	N	19	23	F	N	47
3	M	N	18	12	F	Y	29	24	F	Y	20
4	F	U	18	13	M	N	31	25	M	N	19
5	M	N	34	14	M	Y	29	26	F	Y	37
6	F	Y	47	15	F	Y	37	27	M	N	30
7	F	Y	45	16	M	Y	26	28	M	N	19
8	M	Y	23	17	F	Y	19	29	M	N	28
9	M	Y	18	18	M	Y	42	30	F	N	20
33	F	Y	44	19	F	Y	34	31	M	Y	20
34	M	Y	20	20	F	N	36	32	F	Y	19
35	M	Y	22	21	M	Y	19	69	M	Y	49
36	M	Y	25	51		U	32	70	F	U	26
37	M	Y	32	52	F	N	48	71	F	Y	33
38	F	Y	47	53	F	N	18	72	F	Y	32
39	F	U	19	54	M	Y	27	73	M	Y	33
40	M	Y	21	55	F	N	22	74	F	Y	19
41	M	Y	26	56	F	Y	24	75	F	N	24
42	M	Y	30	57	F	Y	21	76	F	N	22
43	M	Y	26	58	F	Y	35	77	F	Y	42
44	M	N	20	59	F	N	21	78	F	Y	45
45	F	Y	21	60	F	N	26	79	F	Y	23
46	F	Y	42	61	F	N	31	80	M	Y	20
47	F	U	21	62	F	Y	24	81	F	Y	19
48	M	Y	19	63	F	Y	34	82	F	Y	31
49	F	Y	20	64	F	N	21	83	F	N	19
50	F	N	26	65	F	Y	24	84	F	N	20
88	M	Y	32	66	M	Y	37	85	F	N	40
89	F	N	30	67	M	N	20	86	F	Y	30
90	M	N	22	68	M	N	19	87	M	Y	22
91	M	N	24	111	F	Y	37	133	F	N	31
92	M	Y	20	112	F	Y	33	134	M	Y	31
93	M	Y	20	113	F	Y	19	135	M	Y	20
95	M	N	24	114	F	N	21	136	M	Y	20
96	F	N	22	115	M	N	21	137	F	N	21
97	M	Y	21	116	M	Y	26	138	F	N	27
98	F	N	42	117	M	N	28	139	F	Y	19
99	F	N	21	118	F	Y	19	140	F	Y	28
100	M	Y	22	119	M	Y	23	141	F	N	19
101	M	Y	19	120	F	N	27	142	F	N	27
102	M	Y	19	121	M	Y	23	143	F	Y	23
103	F	Y	34	122	M	Y	18	144	F	Y	21
104	M	Y	39	123	F	Y	20	145	F	Y	23
105	F	Y	21	124	F	Y	28	146	F	N	20

106	F	Y	24	125	M	N	20	147	F	Y	21
107	M	Y	20	126	F	Y	18	148	M	Y	22
108	M	U	28	127	M	Y	18	149	M	N	21
109	F	U	21	128	M	Y	36	150	F	Y	20
110	M	N	22	129	M	Y	21	151	F	N	20
				130	M	N	27	152	F	Y	36
				131	M	Y	27				
				132	F	N	21				

Supplemental Table 1. Vaccine comparison study subject demographics. The subject numbers, age, gender and self-reported vaccination status from the previous season are indicated. Vaccination status for the influenza season prior to enrollment was reported as received a vaccine (Y), did not receive vaccine (N), or were unsure (U) if vaccinated last season.
