# Supplementary material for: Recombinant HA-based vaccine outperforms split and subunit vaccines in elicitation of influenza-specific CD4 T cells and CD4 T cell-dependent antibody responses in humans
Source: NPJ Vaccines. 2020 Aug 26;5:77. doi: 10.1038/s41541-020-00227-x (PMC7450042; doi:10.1038/s41541-020-00227-x)
Supplement: Supplementary file 2 — Reporting Summary [file 41541_2020_227_MOESM2_ESM.pdf]

nature research

Corresponding author(s): Andrea Sant

Last updated by author(s): Jun 21, 2020

Reporting Summary

Nature Research wishes to improve the reproducibility of the work that we publish. This form provides structure for consistency and transparency in reporting. For further information on Nature Research policies, see our Editorial Policies and the Editorial Policy Checklist.

Statistics

For all statistical analyses, confirm that the following items are present in the figure legend, table legend, main text, or Methods section.

n/a	Confirmed	
☐	☒	The exact sample size (n) for each experimental group/condition, given as a discrete number and unit of measurement
☐	☒	A statement on whether measurements were taken from distinct samples or whether the same sample was measured repeatedly
☐	☒	The statistical test(s) used AND whether they are one- or two-sided<br>*Only common tests should be described solely by name; describe more complex techniques in the Methods section.*
☐	☒	A description of all covariates tested
☐	☒	A description of any assumptions or corrections, such as tests of normality and adjustment for multiple comparisons
☐	☒	A full description of the statistical parameters including central tendency (e.g. means) or other basic estimates (e.g. regression coefficient) AND variation (e.g. standard deviation) or associated estimates of uncertainty (e.g. confidence intervals)
☒	☐	For null hypothesis testing, the test statistic (e.g. F, t, r) with confidence intervals, effect sizes, degrees of freedom and P value noted<br>*Give P values as exact values whenever suitable.*
☒	☐	For Bayesian analysis, information on the choice of priors and Markov chain Monte Carlo settings
☒	☐	For hierarchical and complex designs, identification of the appropriate level for tests and full reporting of outcomes
☐	☒	Estimates of effect sizes (e.g. Cohen's d, Pearson's r), indicating how they were calculated

Our web collection on statistics for biologists contains articles on many of the points above.

Software and code

Policy information about availability of computer code

Data collection	ELISPOT assays were scored using an Immunospot reader (series 5.2), using Immunospot software, v5.1. For flow cytometry FACS DIVA software (BD Bioscience). Sequential gating was performed using FlowJo version 10 (Tree Star)
Data analysis	Data analysis was completed using FlowJo version 10 and Prism 10.

For manuscripts utilizing custom algorithms or software that are central to the research but not yet described in published literature, software must be made available to editors and reviewers. We strongly encourage code deposition in a community repository (e.g. GitHub). See the Nature Research guidelines for submitting code & software for further information.

Data

Policy information about availability of data

All manuscripts must include a data availability statement. This statement should provide the following information, where applicable:

- Accession codes, unique identifiers, or web links for publicly available datasets
- A list of figures that have associated raw data
- A description of any restrictions on data availability

The data that support the findings of this study are available from the corresponding author upon reasonable request.

Field-specific reporting

Please select the one below that is the best fit for your research. If you are not sure, read the appropriate sections before making your selection.

☒ Life sciences ☐ Behavioural & social sciences ☐ Ecological, evolutionary & environmental sciences

For a reference copy of the document with all sections, see nature.com/documents/nr-reporting-summary-flat.pdf

Life sciences study design

All studies must disclose on these points even when the disclosure is negative.

Sample size	The power analysis is based on the primary aim of comparison of serum antibody, specificity, memory B cell derived polyclonal antibodies (MpAb) and HA-specific CD4 T cell counts (in log scale to stabilize variance) between the group of subjects vaccinated with egg derived TIV IM and the group of subjects vaccinated with Baculovirus derived TIV IM. In this explorative study, the HA-specific CD4 T cell response to the Baculovirus derived vaccine is unknown and thus no information is available about the group difference. But our past experience indicated the coefficient of variation (CV) of HA-specific CD4 T cell counts varied between 0.25 and 0.4. If we assume CV and use percent-change differences in HA-specific CD4 T cell counts relative to the group with egg derived vaccine, with 45 subjects per group , we will have 80% power to detect at least 10% ~ 20% difference in HA-specific CD4 T cell counts between the two groups. This power analysis is based on two-sample t test with a two-sided significance level of 0.05 and equal allocation of subjects to any two groups.
Data exclusions	No data was excluded from the study.
Replication	All subjects were included in the dataset. The methods used have been repeated by our laboratory in numerous studies.
Randomization	Subjects were randomly assigned a vaccine.
Blinding	During data collection and initial analysis all vaccination and demographics information were blinded to the investigators. The groups were unblinded post data collection and initial analysis, when it was appropriate to compare the vaccination groups.

Reporting for specific materials, systems and methods

We require information from authors about some types of materials, experimental systems and methods used in many studies. Here, indicate whether each material, system or method listed is relevant to your study. If you are not sure if a list item applies to your research, read the appropriate section before selecting a response.

Materials & experimental systems

n/a	Involved in the study	
☐	☒	Antibodies
☒	☐	Eukaryotic cell lines
☒	☐	Palaeontology and archaeology
☒	☐	Animals and other organisms
☐	☒	Human research participants
☒	☐	Clinical data
☒	☐	Dual use research of concern

Methods

n/a	Involved in the study	
☒	☐	ChIP-seq
☐	☒	Flow cytometry
☒	☐	MRI-based neuroimaging

Antibodies

Antibodies used	ELISPOT: anti-human interferon (IFN-γ) (clone 1-D1K, MabTech, catalogue number 3420-3-1000) and biotinylated anti-human IFN-γ (clone 7-B6-1, MabTech, catalogue number 3420-6-1000)<br>ELISA: alkaline phosphatase-conjugated anti-human immunoglobulin G (clone MT78, MabTech, catalogue number 3850-9A)<br>Flow Cytometry: Fixable Live/Dead Aqua (Fisher Scientific, catalogue number L34957), CD8 (clone RPA-T8, BD Biosciences, catalogue number 560662), CD4 (clone RPA-T4, BD Biosciences, catalogue number 555346), CD3 (clone SK7, BD Biosciences, catalogue number 560176), CD45RA (clone HI100, BD Biosciences, catalogue number 563733), IL-2 (clone MQ1-17H1BD Biosciences, catalogue number 564165), IFN-γ (clone B27, BD Biosciences, catalogue number 562988), CD69 (clone FN50, BD Biosciences, catalogue number 555532) and CD19 (clone HIB19, Biolegend catalogue number 302236)
Validation	The antibodies have been validated by the manufacturer and have been used in multiple publications both by our laboratory and other investigators.

Human research participants

Policy information about studies involving human research participants

Population characteristics	Study subjects aged 18 to 49 years old were randomized into three cohorts, each receiving a licensed seasonal influenza vaccine. The vaccine formulations administered were egg-derived split-vaccine Fluzone quadrivalent (blue), mammalian cell-based Flucelvax (gold), recombinant HA made in the baculovirus system Flublok (red) and egg-derived split-vaccine Fluzone High (HD) dose (light blue). Subjects were enrolled for a single season in one of the vaccine groups over. Over the course of 3 consecutive seasons 150 healthy subjects were divided into the first three vaccine groups as indicated in the left-hand portion of the Figure. In the third season, an additional 21 subjects were enrolled in the Fluzone HD group. Blood samples were taken at day 0 (D0) prior to vaccination, and day 7 (D7, for flow cytometry, which peaks for some subsets of CD4 cells at this time), day 14 (D14, for EliSpot assays that peak at this time point) and day 28 (D28, neutralizing antibody) post-vaccination. Sera for HAI assays were sampled at day 0 and day 28.
Recruitment	Up to 500 subjects will be enrolled from the existing population of healthy adults residing in Rochester, NY and within the University of Rochester community, males and non-pregnant females aged 18-49 years, inclusive. Pregnancy status will be verified at screening and enrollment prior to vaccination. Women of childbearing potential will be asked to use a barrier method of birth control or an FDA approved form of birth control.<br>Study subjects will be recruited via placement of flyers in key locations on campus. Based on previous experience, we anticipate enrollment of 40 to 60 percent non-pregnant females in the study.
Ethics oversight	Division of Microbiology and Infectious Diseases (DMID) and the UR Research Subjects Review Board (protocol l5-0055)

Note that full information on the approval of the study protocol must also be provided in the manuscript.

Flow Cytometry

Plots

Confirm that:

☐ The axis labels state the marker and fluorochrome used (e.g. CD4-FITC).

☐ The axis scales are clearly visible. Include numbers along axes only for bottom left plot of group (a 'group' is an analysis of identical markers).

☐ All plots are contour plots with outliers or pseudocolor plots.

☒ A numerical value for number of cells or percentage (with statistics) is provided.

Methodology

Sample preparation	PBMC from pre-vaccination (day 0) and day 7 post-vaccination were thawed, rested in culture overnight, washed, depleted of CD69+ cells using MACS microbeads (Miltenyi Biotec), to eliminate cells expressing the activation marker, and stimulated with the peptide pools for 16hours. In the 200 ul culture, 2 ul of Brefeldin A and 4 ul of Monensin (1:100) were added or the last 8hours of stimulation (BD Biosciences, catalogue numbers 555029 and 554724, respectively). Cells were washed and stained for 30minutes with Fixable Live/Dead Aqua. Surface and intracellular staining was performed as previously described 70,71. Antibodies included CD8 (RPA-T8), CD4 (RPA-T4), CD3 (SK7), CD45RA (HI100), IL-2 (MQ1-17H1), IFN-γ (B27), CD69 (FN50) (BD Biosciences) and CD19 (HIB19) (Biolegend), with cell fixation and permeabilization performed using the eBioscience FoxP3 cytofix/perm kit. Fluorochrome-stained UltraComp e-Beads (Invitrogen) or ArC Amine Reactive Compensation Beads (ThermoFisher Scientific) were used for compensation.
Instrument	Data was acquired using a BD LSR-II instrument, configured with 488, 633, 407, and 532-nm lasers using FACS DIVA software (BD Bioscience).
Software	FACS DIVA software (BD Bioscience). Sequential gating was performed using FlowJo version 10 (Tree Star)
Cell population abundance	*Describe the abundance of the relevant cell populations within post-sort fractions, providing details on the purity of the samples and how it was determined.*
Gating strategy	Sequential gating was performed using FlowJo version 10 (Tree Star). Lymphocytes were first gated on forward and side scatter, followed by singlet gating and then live cells. Activated cells of interest were defined as CD69+ CD4+ T cells (CD3+, CD8−) that were positive for cytokine staining (IFN-γ and/or IL-2).

☒ Tick this box to confirm that a figure exemplifying the gating strategy is provided in the Supplementary Information.
